# Supplementary material for: Anoxic Treatment of Agricultural Drainage Water in a Venturi-Integrated Membrane Bioreactor
Source: Membranes (Basel). 2023 Jul 14;13(7):666. doi: 10.3390/membranes13070666 (PMC10385815; doi:10.3390/membranes13070666)
Supplement: Supplementary file 1 [file membranes-13-00666-s001.zip › S1 H2 Gas Efficiency Calculations.pdf]

Supplementary Material S1 – H2 Gas Efficiency Calculations

Measurements/assumptions

1) Removed NO_3-N leaves the system as N_2 gas.
2) Hydrogen gas is consumed only in denitrification. (This assumption is acceptable since nitrate-added tap water was used as feedwater when determining the H_2 gas utilization efficiency.)
3) 0.357 mg of H_2 gas is used to remove 1 mg of NO_3-N. (Eqn. 1)
4) The ambient temperature is 25 °C.
5) The inlet NO_3-N concentration is $C_{NO3} = 102\frac{mg}{L}$, and effluent NO_3-N and NO_2-N concentrations are 9.3 mg/L and 2.3 mg/L, respectively.
6) Wastewater feed discharge is $Q = 10.6\ mL/min$ (15313 L of permeate was collected in a day)
7) The volume of the gas collection bag is 2 L.

The partial pressures of N_2 and H_2 gases were measured as 76.6 and 17%, respectively at the headspace gas collected.

The mass of NO_3-N removed daily is found as follows.

$$m_{N_2} = Q \cdot C_{N_2} \cdot t = 10.6\ \frac{mL}{min} \cdot 10^{-3}\frac{mL}{L} \cdot (102 - 9.3 - 2.3)\ \frac{mg}{L} \cdot \left(24 \cdot 60\ \frac{min}{day}\right)$$
$$= 1379.9\ \frac{mg\ NO_3 - N}{day}$$

Here, m_{N_2} denotes mass of nitrogen gas, Q permeate discharge, C_{N_2} influent concentration of nitrate nitrogen, and t time.

The mass of H_2 used for daily NO_3-N removal is found as follows.

$$m_{H_2}^{(1)} = 0.357 \cdot m_{N_2} = 0.357\ \frac{mg\ H_2}{mg\ NO_3 - N} \cdot 1379.9\ \ mg\ NO_3 - N = 492.6\ mg$$

Since the molecular weight of nitrogen gas is $M_{N_2} = 28\ \frac{mg}{mmol}$, the mole equivalent of NO_3-N removed is found as follows:

$$n_{N2} = \frac{1379.9\ mg}{28\ \frac{mg}{mmol}} = 49.3\ mmol$$

The partial pressure of this amount of gas at 25 °C according to ideal gas law, $PV = nRT$, is calculated as follows.

$$p_{N_2} = \frac{49.3\ mmol \cdot 10^{-3}\ \frac{mol}{mmol} \cdot 8.314\ \frac{J}{mol \cdot K} \cdot (273 + 25)K}{2\ L \cdot \frac{1\ m^3}{1000\ L}} = 61072\ Pa$$

Since the partial pressure of nitrogen gas is assumed to be 17%, the total gas pressure is found as follows:

$$p_T = \frac{61072\ Pa}{0.766} = 79729\ Pa$$

Because the partial pressure of H_2 gas is 17%, its pressure is found as follows:

$$p_{H_2} = p_T \cdot 0.766 = 79729 \cdot 0.17 = 13554\ Pa$$

Using this pressure, the mole of H_2 is calculated from ideal gas law.

$$n_{H_2} = \frac{p_{H_2} \cdot V}{RT} = \frac{13554 \cdot 2\ L \cdot \frac{1\ m^3}{1000\ L}}{8.314\ \frac{J}{mol \cdot K} \cdot (273 + 25)K} = 0.0109\ mole$$

The mass of hydrogen gas corresponding to this amount of mole is found as follows:

$$m_{H_2}^{(2)} = n_{H_2} \cdot M_{H_2} = 0.0109\ mol \cdot 2\ \frac{g}{mol} = 0.0219\ g = 21.9\ mg$$

The mass of hydrogen gas supplied to the reactor is calculated by adding together the mass of H_2 gas used for denitrification and the mass of the unused hydrogen gas.

$$m_{H_2}^{T} = m_{H_2}^{(1)} + m_{H_2}^{(2)} = 492.6 + 21.9 = 514.5\ mg$$

The H_2 gas utilization efficiency is calculated by dividing the amount gas used by the total amount supplied to the reactor.

$$\eta_{H_2} = 100 \cdot \frac{hydrogen\ utilized}{total\ hydrogen\ supplied} = 100 \cdot \frac{492.6\ mg}{514.5\ mg} = 95.7\ \%$$
