# Supplementary material for: Anoxic Treatment of Agricultural Drainage Water in a Venturi-Integrated Membrane Bioreactor
Source: Membranes (Basel). 2023 Jul 14;13(7):666. doi: 10.3390/membranes13070666 (PMC10385815; doi:10.3390/membranes13070666)
Supplement: Supplementary file 1 [file membranes-13-00666-s001.zip › S2 Photos of Experimental Setup.pdf]

Supplementary Material S2 – Photos of Experimental Setup

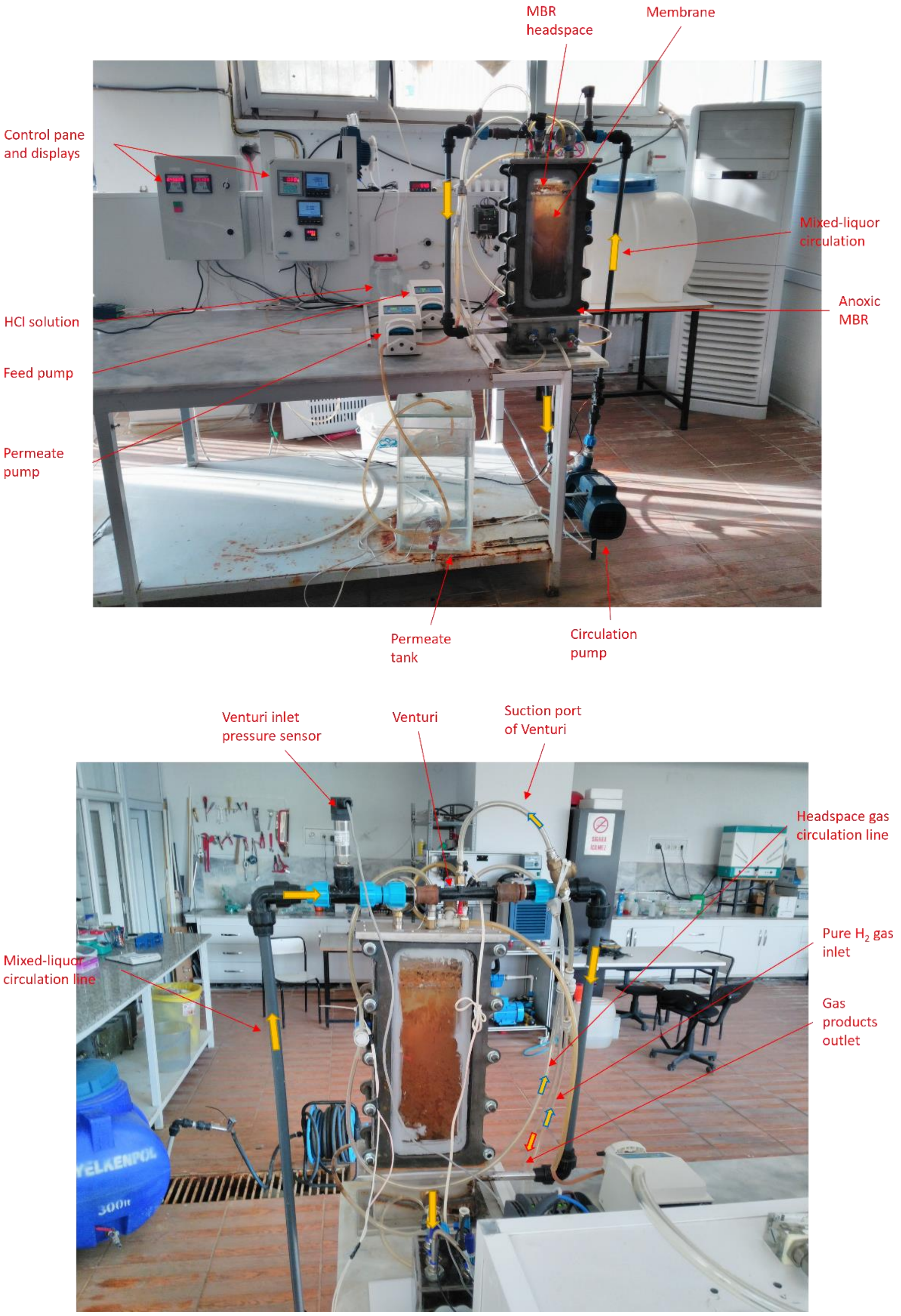

Experimental setup: front view (upper) and back view (lower) (Gul and Kayaalp, 2022)
