# Supplementary material for: Anoxic Treatment of Agricultural Drainage Water in a Venturi-Integrated Membrane Bioreactor
Source: Membranes (Basel). 2023 Jul 14;13(7):666. doi: 10.3390/membranes13070666 (PMC10385815; doi:10.3390/membranes13070666)
Supplement: Supplementary file 1 [file membranes-13-00666-s001.zip › S3 FTIR Graphs.pdf]

Supplementary Material S3 – FTIR Graphs

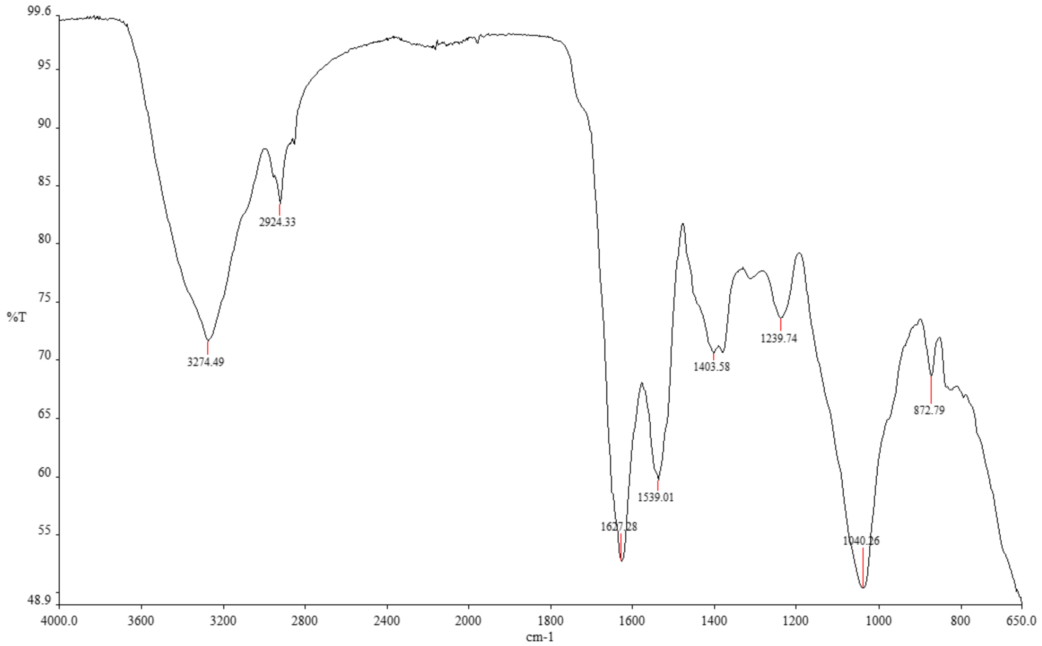

(a)

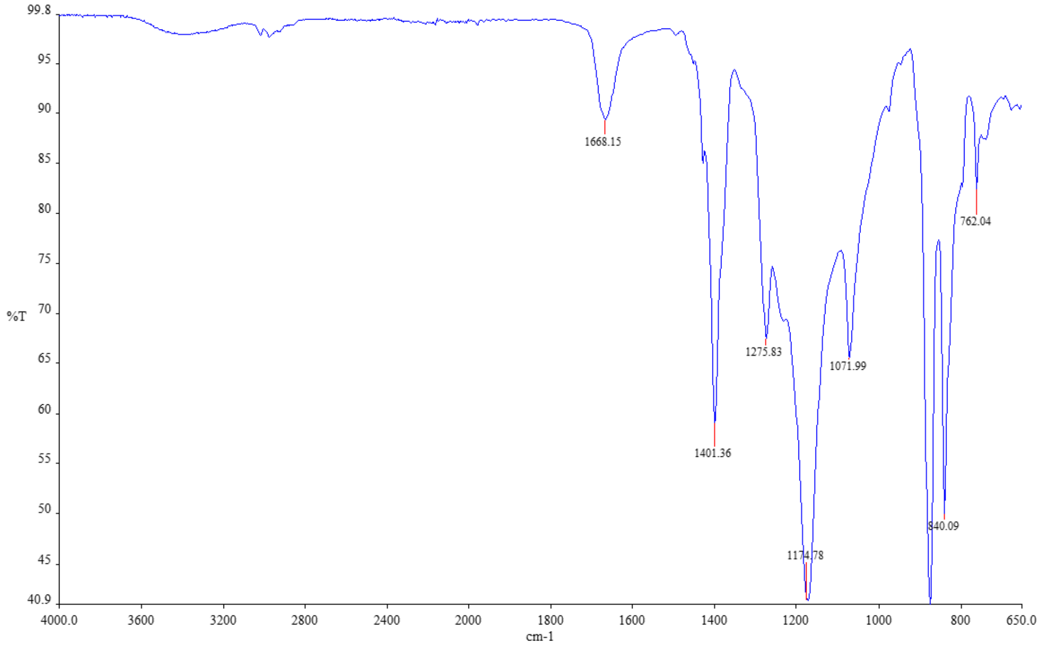

(b)

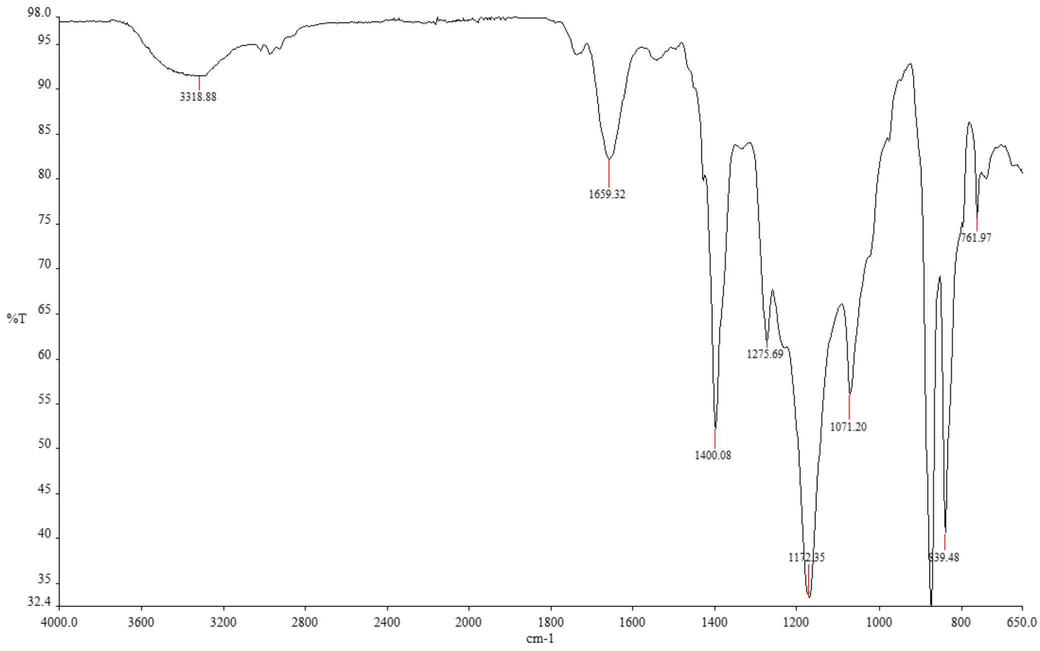

(c)

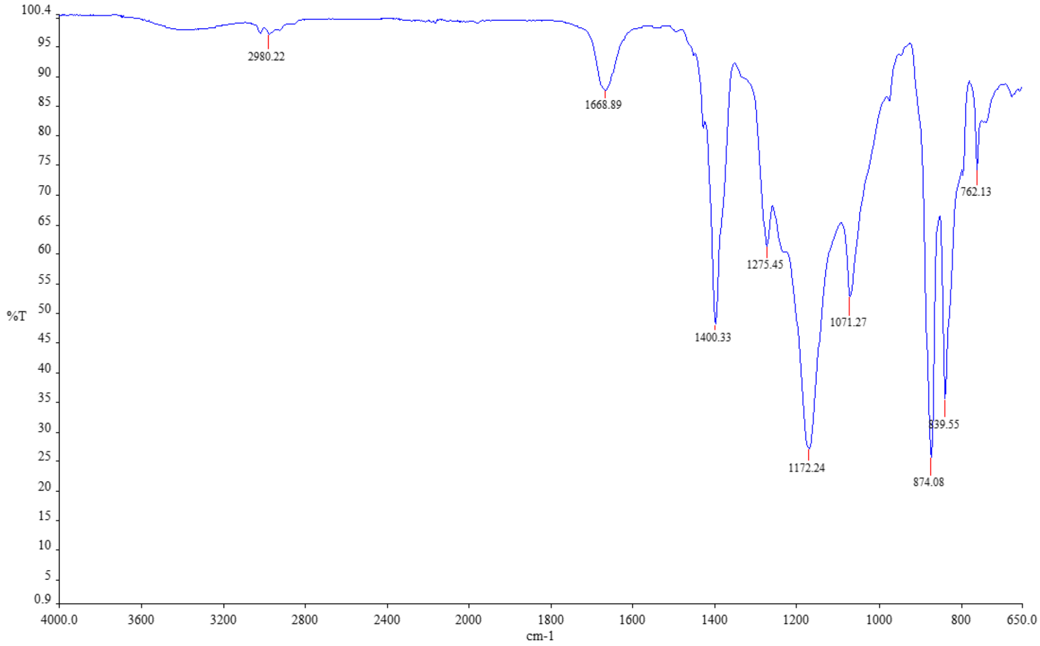

(d)

FT-IR graph of fouled (a) and cleaned (b) membranes in configuration using venturi without headspace gas circulation, and of fouled (c) and cleaned (d) membranes in configuration using venturi with headspace gas circulation.
