# Supplementary material for: Anoxic Treatment of Agricultural Drainage Water in a Venturi-Integrated Membrane Bioreactor
Source: Membranes (Basel). 2023 Jul 14;13(7):666. doi: 10.3390/membranes13070666 (PMC10385815; doi:10.3390/membranes13070666)
Supplement: Supplementary file 1 [file membranes-13-00666-s001.zip › S4 SEM Images.pdf]

Supplementary Material S4 – SEM Images

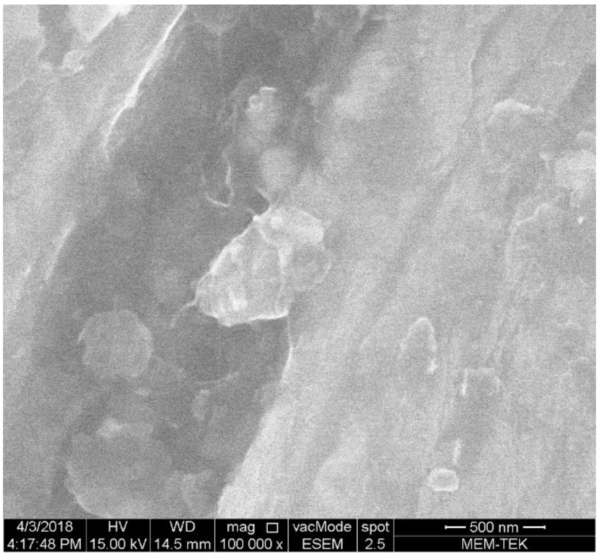

(a)

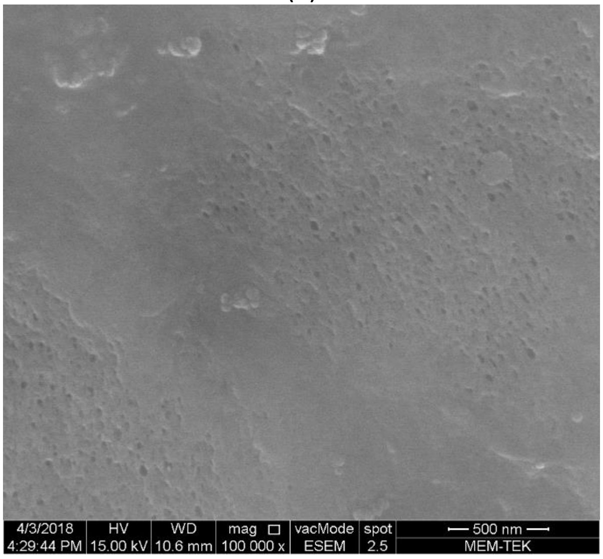

(b)

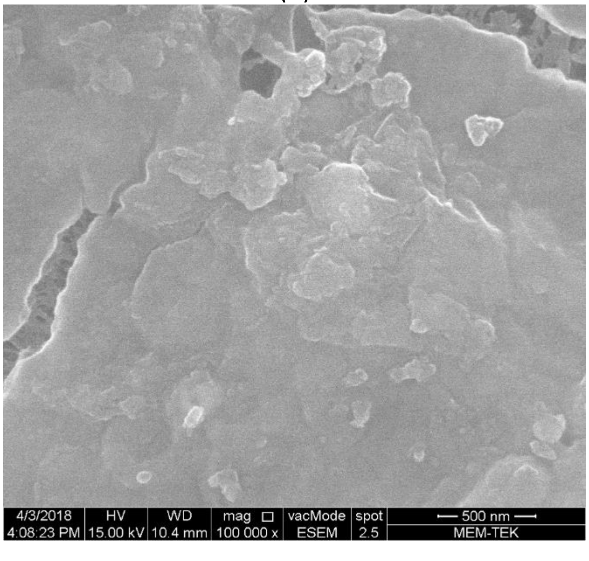

(c)

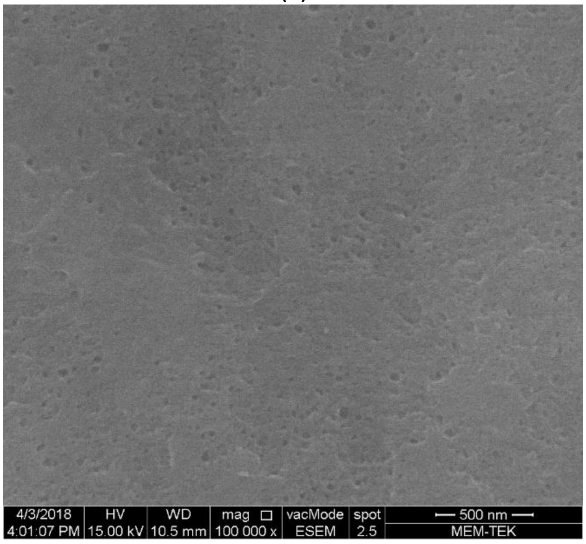

(d)

Figure 11: SEM images of fouled (a) and cleaned membrane (b) in the absence of headspace gas circulation using the venturi device and fouled (c) and cleaned (d) membrane in case of headspace gas circulation using the venturi device (magnification:100 000 x).
